# Chemokine Contribution in Stem Cell Engraftment into the Infarcted Myocardium

**DOI:** 10.2174/1574888X11308040003

**Published:** 2013-07

**Authors:** Elisa A Liehn, Eugen Radu, Alexander Schuh

**Affiliations:** 1 Institute for Molecular Cardiovascular Research (IMCAR), RWTH Aachen University, Germany;; 2 Department of Cellular and Molecular Medicine, ‘Carol Davila’ University of Medicine and Pharmacy, Bucharest, Romania;; 3 Department of Cardiology, Pneumology, Angiology and Internal Medicine Intensive Care (Internal Medicine I), University Hospital, RWTH Aachen University, Germany

**Keywords:** Cell transplantation, chemokine, inflammation, myocardial infarction, remodeling.

## Abstract

Modern life styles have made cardiovascular disease the leading cause of morbidity and mortality worldwide. Although current treatments substantially ameliorate patients’ prognosis after MI, they cannot restore the affected tissue or entirely re-establish organ function. Therefore, the main goal of modern cardiology should be to design strategies to reduce myocardial necrosis and optimize cardiac repair following MI. Cell-based therapy was considered a novel and potentially new strategy in regenerative medicine; however, its clinical implementation has not yielded the expected results. Chemokines seem to increase the efficiency of cell-therapy and may represent a reliable method to be exploited in the future. This review surveys current knowledge of cell therapy and highlights key insights into the role of chemokines in stem cell engraftment in infarcted myocardium and their possible clinical implications.

## INTRODUCTION

Acute myocardial infarction (MI) continues to be the leading cause of morbidity and mortality worldwide despite considerable efforts and numerous advances in the diagnosis and management of the disease. In light of increasing stress levels resulting from massive expansion of information traffic, we can expect the future to bring an increased incidence of cardiovascular disease and myocardial infarction in human populations. Therefore, developing new therapeutic strategies for preventing and even curing atherosclerosis and its complications, such as MI or stroke, must be a priority for the scientific community.

Due to the modern predominance of sedentary yet stressful lifestyles, atherosclerosis, the deterioration of arterial walls as a result of the accumulation of cholesterol, is the most common disease afflicting humans worldwide. This accumulation of cholesterol is followed by a severe thickening and other changes in vessel morphology, including narrowing of the vessel lumen [1]. In this context, the high shear stress that develops in the narrowing arteries can cause the luminal endothelium to rupture and release its lipid content into the vessels, which in turn determines the local accumulation of thrombotic material and obstruction of blood flow. In the case of coronary arteries, which do not develop collaterals, the acute reduction in blood flow cannot be properly compensated [2]. Given the greater oxygen demands of the myocardium, this means that even a short interruption to blood flow here can have serious consequences. Damaged cardiac tissue is replaced by granulation tissue that later matures into a scar [3], which in turn induces global changes in heart architecture with progressive dilatation and, ultimately, development of heart failure. Therefore, beyond acute mortality, MI results in subsequent complications that reduce patients’ quality of life.

Because the molecular mechanisms are poorly understood, it is difficult to develop new therapeutic strategies. However, it seems that the chemokines play an important role in initiating and controlling events at the molecular level during pathologic processes [4].

Chemokines are a glycoprotein family with chemotactic activity in concert with a subfamily of G-protein coupled seven-transmembrane receptors [5]. Depending on the position of the N-terminal cysteines, chemokines are classified into four subfamilies: CXC chemokines (containing a single amino acid between the first and second cysteine residues), CC chemokines (adjacent cysteine residues), the C chemokine group (called lymphotactin, which lacks one of the cysteines), and CX3C chemokine (called fractalkine/neurotactin, with three amino acid residues between the first two cysteines).

In atherosclerosis, more than 40 chemokines, signaling through almost 20 receptors, initiate and modulate leukocyte trafficking [2]. For example Platelet Factor 4 (CXCL4) or RANTES (CCL5), released from the activated platelets, both increase monocyte arrest onto inflamed endothelium [6, 7]. Fraktalkine (CX3CR1) and KC (CXCL1) determine firm monocyte adhesion *via* CX_3_CR1 [8] and CXCR2 [9], while their transmigration is accomplished *via* the MCP-1 (CCL2)/CCR2 pathway [10]. SDF-1 (CXCL12) and its receptors CXCR4 and CXCR7 [11] control stem cell trafficking, smooth muscle cell proliferation [12] and endothelial cell migration and angiogenesis [13].

Present therapies are limited to cause-dependent interventions such as decreasing blood pressure and cholesterol, treating diabetes mellitus, or balloon dilatation and stent implantation. Although these treatments substantially ameliorate a patient’s prognosis after MI, they do not restore affected tissue or entirely re-establish organ function. Therefore, the main goal of modern cardiology should be to design strategies to reduce myocardial necrosis and optimize cardiac repair following MI.

Cell-based therapy is a novel strategy considered to have great potential in regenerative medicine. The ultimate goal of stem cell-based cardiac repair is the regeneration of healthy, functionally integrated myocardial tissue. But in spite of encouraging results from experimental studies, cell-therapy has not yielded satisfying results in clinical implementation, probably due to the difficulties of translating knowledge from animal to human systems. Therefore, in order to create efficient therapies, we urgently need to understand how each of these processes is modulated and controlled. 

## CELL-BASED THERAPIES: PRESENT AND FUTURE

Cell-therapy refers to the use of cells to treat diseases. Modern cell-based therapy has advanced dramatically from the first human-human blood transfusion, almost 200 years ago, to bone-marrow and organ transplantation, tissue banking and reproductive *in vitro* fertilization today. In recent decades, stem cells and adult cells derived from various types of tissues have been isolated, characterized and cultivated *in vitro*, and the technique has proven beneficial not only to animals, but also to humans in the treatment of diverse diseases.

As a potential new strategy in the treatment of cardiovascular disease, cell-based therapy has seemed very promising from the outset. After MI, stem cells are expected to integrate at the site of injury, replace damaged cardiomyocytes and vessels [14], and thereby restore the initial integrity of the myocardium and facilitate preservation of heart function [15]. In trying to elucidate the basic mechanisms, experimental studies have used many types of cells, including bone-marrow derived stem cells [16-19], mesenchimal stem cells (MSCs) [20, 21], fetal cardiomyocytes [22, 23], and even angiogenic progenitors such as endothelial progenitor cells (EPCs) [24] or human umbilical vein endothelial cells (HUVECs) [25]. Intramyocardial application of these cells immediately after MI in animal models seems to have the most pronounced effects, but systemic transplantation has shown benefits as well [26-29]. Unfortunately, despite extensive research over the past few years, clinical studies have not yielded the results researchers have hoped for, and an understanding of the cell-mediated regenerative mechanisms remains elusive [30]. As a result it is still impossible to implement cell therapy in current clinical practice, and it therefore remains imperative to find new methods to sustain and improve the effects of cell therapy. 

Recent observations in this context have shown that chemokines are directly involved in the trafficking and integration not only of endogenous, but also of transplanted stem cells [4, 31]. It is already known that chemokines interfere with key events following MI: they modulate the inflammatory response, the molecular and cellular composition of the scar, and the implicit remodeling of the ventricle and heart function [3]. 

The chemokines are strongly upregulated after MI and are responsible for the initiation of the inflammatory and reparatory processes, including stem cell recruitment and engraftment into the myocardium [3, 32]. For example, after MI chemokines such as CCL2 [33], CXCL12 [34] or Macrophage Inhibitory Factor (MIF) [35] are upregulated and exert their cardioprotective function. Neutrophil recruitment is initiated and sustained by CCL3 and CCL5 chemokines (*via* CCR1 [36] and CXCL12 [37]) followed by inflammatory monocyte infiltration [38] (regulated by the CCL2/CCR2 pathway [39]), which is responsible for clearing the wound of cellular debris. The reparatory monocytes (using CX3CL1/CX3CR1 pathway [39] or CCR5 [40]) promote subsequent healing *via* myofibroblast accumulation, angiogenesis, and deposition of collagen. SDF-1α/ CXCL12 also interfere during this phase, where they sustain and improve angiogenesis with beneficial effects on myocardial remodeling and cardiac function [41, 42]. However, the upregulation of angiogenic factors like SDF-1α/CXCL12, GROα/CXCL1 [43], IL8/CXCL2 [44], MIF [45, 46] or MCP1 [47] after MI is countered by the release of angiostatic chemokines such as CXC chemokine interferon-γ inducible protein (IP)-10 [48], whose role is to inhibit angiogenesis until the myocardium has been cleaned of cellular debris and a provisional matrix has formed, which in turn supports the growth of new blood vessels [48, 49]. Recently, CCR5-mediated regulatory T cells have been found to restrain post-infarction inflammation, thereby preventing excessive matrix degradation and attenuating adverse remodeling [50]. Finally, chemokines like SDF-1α [42, 51] and MCP-3 [52] control myocardial homing of Mesenchimal Stem Cells (MSCs) after injury, which in turn facilitates the replacement of dead cardiomyocytes and the regeneration of heart tissue. 

Therefore, the use of chemokines to increase the efficiency of cell-therapy seems a promising and potentially reliable method that ought to be exploited. This review surveys current knowledge about the contribution of chemokines in processes of stem cell engraftment into infracted myocardium and highlights its possible clinical implications. 

## CXC CHEMOKINES AND CELL TRANSPLANTATION

One of the most important effects of cell-therapy is considered to be increased angiogenesis. Since an appropriate level of blood perfusion is able to assure and sustain endogenous processes of regeneration, many efforts have concentrated on studying the complex mechanisms of angiogenesis involving progenitor cells and angiogenic factors. Endothelial progenitor cells (EPCs) are responsible for optimal formation of new vessels and vascularization in the infarcted areas [53], and are mostly recruited and controlled by vascular endothelial growth factor (VEGF) and stromal derived factor (SDF)-1α/CXCL12 [54, 55]. Whereas VEGF was unable to add to improved heart function after transplantation of fetal cardiomyocytes [22] in a rat model of MI, SDF-1α/ CXCL12 has been used widely in different experimental settings both *in vivo* and *in vitro* and found to amplify the effect of cell-transplantation [24, 41]. As we know, angiogenesis takes place over the entire proliferation and healing phase; SDF-1α/CXCL12 synthesis, however, shows a peak shortly after the hypoxic insult induction in animal models [56]. Therefore, an external therapeutic transplantation of EPCs in a human model at this juncture would probably have maximally beneficial effects as compared to a later, systemic transplantation of EPCs after MI. 

SDF-1α/CXCL12 plays the key role in hemato-, vasculo- and cardiogenesis [57-59]. However, it became the most studied chemokine in the cardiovascular field due to its capacity to promote the migration not only of stem cells [54], endothelial progenitor cells (EPCs) [37], and smooth muscle cells [12], but also of mesenchimal stem cells (MSCs) [60]. The mechanisms of myocardial protection or regeneration conferred by the CXCL12/Cxcr4 axis may involve both recruitment of circulating cells and effects on resident cardiomyocytes. Cardioprotective SDF-1α/CXCL12 activates the cell-survival factor protein kinase B (PKB/Akt) *via* Cxcr4 and protects ischemic myocardium. This decreases scar size and mediates neovascularization in mice and rats [24, 34]. The interaction between SDF-1α/CXCL12 and Cxcr4 has been increasingly exploited in an effort to enhance the efficacy of stem cell therapy after MI [42, 51]. Exogenous application of SDF-1α/CXCL12 locally after MI in a mouse model has been found by itself to improve remodeling and heart function [61] by inducing angiogenic/progenitor cell homing and increasing capillary density. Moreover, overexpressing SDF-1α/CXCL12 in EPCs before transplantation has resulted in a significant improvement over a normal EPC group, in both LV function and angiogenesis, following intramyocardial application in rats [29, 62]. 

Mesenchymal stem cells (MSCs) have also been described as cardiac precursors [20, 63, 64]. They are found mostly in bone marrow [65, 66], but MSC-like cells have also been described in adipose tissue [67], dental pulp [68], fetal liver [69], fetal lung [70] and in the umbilical cord [71]. These cells seem to play an important role in cardiac regeneration after injury in animal models [72, 73], and, due to their capacity to differentiate into a variety of cell types depending on environmental conditions, they have been widely used as a source of stem cells for therapy after myocardial infarction [20, 21].

Mesenchymal stem cells (MSCs) also secrete SDF-1α/CXCL12, which, reacting with its receptor CXCR4, increases their survival. Intravenous administration of SDF-1α/CXCL12-overexpressing MSCs one day after MI induction in a rat model has shown increased cardiac myocyte survival and vascular density within the infarct zone as compared to transplantation of normal MSCs [42]. In the same model, over-expression of SDF-1α/CXCL12-ligand CXCR4 in MSCs enhanced mobilization and engraftment of MSCs into ischemic areas *in vivo*, in turn promoting neoangiogenesis and left ventricular remodeling [51]. 

The mechanisms responsible for angiogenesis are very complex and involve many more chemokines than SDF-1α/CXCL12. Among these are GROα/CXCL1 [43], IL8/CXCL2 [44], macrophage inhibitory factor (MIF) [45, 46] and even MCP1 [47]. In addition to their role in angiogenesis, these factors seem to influence the course of cell-based therapy in an important way. GROα/CXCL1 plays an important role in preventing damage to viable myocardium and in preserving heart function after transplantation of autologous bone marrow-derived mononuclear cells following MI in a rat model [74]. IL8/CXCL2 [75] can prolong the residence time of MSCs injected into rat myocardium following MI. Macrophage migration inhibitory factor (MIF) is a pleiotropic inflammatory cytokine with chemokine-like functions. It has recently been shown to bind CXCR2 and CXCR4 *in vitro *[76]. Beyond its role in angiogenesis, MIF is a very important factor in the recruitment and chemotaxis of EPCs both *in vitro *[45] and *in vivo *[53]. It acts mostly upon hypoxic conditions in a CXCR4-dependent manner. However, its role in cell-transplantation is far for being understood.

Many other CXC chemokine receptors are expressed on bone-marrow derived MSCs, for instance CXCR3 or CXCR6; but their role in cell-based therapy is so far unknown [77]. 

## CX3C CHEMOKINES AND CELL TRANSPLANTATION

Other chemokines such as CCL25 or CX3CL1 have been shown to modulate MSC chemotaxis, not only *in vitro *[78] but also *in vivo*, in a rat model of brain ischemia [79]. In myocardial infarction, CX3CR1 is responsible for the trafficking of reparatory monocytes and regeneration [39]. However, its precise role following cell therapy is not known.

## CC CHEMOKINES AND THE CELL-TRANSPLANTATION

Beside CXC chemokines, CC chemokines have also proved important in cell engraftment and remodeling after cell-therapy. CCL2, known as monocyte chemotactic protein 1, is up-regulated after MI in animal models [80], and is important in angiogenesis and collateralization both *in vivo* and *in vitro *[81, 82]. However, some data have established that CCL2 plays a role in collateralization and in the homing of MSCs to the ischemic myocardium in both animal models and in patients [81, 82]. Recently, CCL7, also known as monocyte chemotactic protein 3, has been found to be crucially involved both in MSCs homing to the ischemic myocardium as well as in their intramyocardial migration and survival [52]. Moreover, overexpression of CCL7 induced homing of repeatedly administrated MSCs as long as one month after MI in a rat model, which in turn improved heart remodeling and preserved heart function [52]. Beside CCR2, CCR1 has been found to bind CCL7 [83], thereby increasing the recruitment of MSCs and protecting them from apoptosis after transplantation at the infarction site in a murine model [83].

Unfortunately, in spite of evidence that the chemokine and chemokine receptor are expressed on progenitor cells and are important in their trafficking, there is only limited data on their role and potential for use in the cell therapy.

## CONCLUSION

In conclusion, there is a great deal of evidence indicating that chemokines are crucial in myocardial migration, engraftment and survival of various types of stem cells after transplantation. Tasks including specific recruitment at the injury site, establishment of connections with surrounding cells, and other specific functions of transplanted stem cells can only take place under close monitoring by the chemokines. 

However, despite extensive research in recent years, the cell-mediated regenerative mechanisms remain elusive, rendering it impossible to implement cell therapy in current clinical practice. In light of this, new therapeutic strategies must be found in order to improve heart regeneration and remodeling following MI. Though little studied, chemokines may be the key molecules in modulation, control and improved survival and integration of transplanted cells at the site of myocardial infarction. Extensive research is still necessary to establish the exact role of each chemokine and any ways one or more of them may be used to improve the cell-based therapy.
